# Predicting ADHD symptoms and diagnosis at age 14 from objective activity levels at age 7 in a large UK cohort

**DOI:** 10.1007/s00787-020-01566-9

**Published:** 2020-06-06

**Authors:** Valerie Brandt, Praveetha Patalay, Julia Kerner auch Koerner

**Affiliations:** 1 Department of Psychology, Center for Innovation in Mental Health, University of Southampton, Southampton, Hampshire UK; 2 Centre for Longitudinal Studies, Institute of Social Research, UCL, London, UK; 3 Faculty of Population Health Sciences, MRC Unit of Lifelong Health and Ageing, UCL, London, UK; 4 Educational Psychology, Helmut-Schmidt-University Hamburg, Holstenhofweg 85, 22043 Hamburg, Germany; 5 Center for Individual Development and Adaptive Education of Children at Risk (IDeA), Frankfurt, Germany

**Keywords:** Attention deficit hyperactivity disorder (ADHD), Actigraph, Accelerometer, Activity, Longitudinal

## Abstract

Hyperactivity is one of the three core symptoms in children with attention deficit hyperactivity disorder (ADHD). Diagnosing ADHD typically involves self-report, third party report and observations. Objective activity data can make a valuable contribution to the diagnostic process. Small actigraphy studies in clinical samples have shown that children with ADHD move more than children without ADHD. However, differences in physical activity between children with and without ADHD have not been assessed in large community samples or longitudinally. This study used data from the Millennium Cohort Study to test whether symptoms of ADHD (parent-rating Strengths and Difficulties Questionnaire) and ADHD diagnosis at age 14 (reported by parents) could be predicted from objective activity data (measured with actigraphs) at age 7 in *N* = 6675 children (final *N* = 5251). Regressions showed that less sedentary behavior at age 7 predicted more ADHD symptoms at age 14 (*β* =  − 0.002, CI  − 0.004 to  − 0.001). The result remained significant when controlled for ADHD symptoms at age 7, sex, BMI, month of birth, SES and ethnicity (*β*  =  − 0.001, CI  − 0.003 to  − 0.0003). ADHD diagnosis at age 14 was also significantly predicted by less sedentary behavior at age 7 (*β*  =  − 0.008). Our findings show that symptoms of ADHD can be predicted by objective activity data 5 years in advance and suggest that actigraphy could be a useful instrument aiding an ADHD diagnosis. Interestingly, the results indicate that the key difference between children with and without ADHD lies in reduced sedentary activity, i.e., times of rest.

**Electronic supplementary material:**

The online version of this article (10.1007/s00787-020-01566-9) contains supplementary material, which is available to authorized users.

## Background

Attention deficit hyperactivity disorder (ADHD) is a common childhood-onset, neurodevelopmental disorder that is characterized by three core symptom domains: hyperactivity, inattention and impulsivity [1]. ADHD is a multifaceted disorder with a prevalence of 5% [2]. ADHD is characterized by persistent and trans-situational hyperactivity/impulsivity, inattention or both with a childhood onset and an impairment in functioning [1].

To diagnose ADHD, clinicians typically rely on observations and reports from several different sources, such as the parents, school reports and the report of the affected child or adult. However, one of the main limiting factors in reaching an ADHD diagnosis is limited, corroborating evidence from family and friends [3]. Neuropsychological tests, such as continuous performance tests, can also be conducted but are less commonly used than observation [3, 4]. The subjectivity of observations and reports, and the absence of a ‘gold standard’ diagnostic process has been criticized [4, 5]. The diagnostic process is currently subjective, rather lengthy and there is no internationally agreed upon recommendation about which combination of measures should be used [4].

A few objective measures can be used to aid the diagnostic process and assess symptom domains in ADHD, such as continuous performance tests and actigraphy. Continuous performance tests can assess attention and impulsivity and could potentially aid a clinical diagnosis, although evidence is somewhat mixed [4]. The usefulness of continuous performance tests can be improved by assessing activity levels in addition [6, 7]. The QbTest (Qbtech Ltd), for instance, uses infrared motion analysis during a computerized continuous performance tests to detect motor activity during the test and has been developed to augment clinical diagnosis [8, 9]. While the Qb test assesses activity in a clinical setting, actigraphs can assess activity levels objectively in everyday life and can record long stretches of time.

Actigraphy is a non-invasive, objective measure of physical activity. Actigraphs are worn on the body and record motor activity. A number of smaller studies have found that children with ADHD have a higher activity level than controls [10–13], while one study found no group difference [14]. Children with ADHD displayed higher levels of activity during a day of clinical assessment compared to control children [10]; they showed increased activity compared to control children during 3 h in school, particularly in art class [11], and during a stop-signal task, a choice task and a control task [12]. Children with ADHD were also more active than psychiatric patients without ADHD and healthy controls [13]. Twelve boys with ADHD were given dextroamphetamines on and off for 4 weeks and were monitored 24 h a day. The results show decreased activity levels under dextroamphetamines compared to placebo during daytime and increased levels during nighttime [15]. Differences in activity levels across the different ADHD subtypes were not found [16]. For a review see [17]. Adolescents, who had been diagnosed with ADHD in childhood, showed more activity than controls independent of whether they still met criteria for ADHD or not [18].

A line of studies has shown that increased activity level ratings in infancy positively predict ADHD symptoms in later childhood [19–22]. Studies using objective measures did not find that movement-related parameters in infancy predicted ADHD in childhood [20, 23]. It was suggested that this may be due to the short time frame these measures captured on a single day [20] and data was recorded in an unfamiliar, experimental setting. Furthermore, different movement parameters that can be inferred from actigraphy data, such as the amount of sedentary activity or time spent in vigorous activity, have not been explored [24].

Moreover, objective measures of ADHD indicators can be helpful in assessing outcomes for intervention, in addition to ratings. For instance, a study using actigraphy data showed no beneficial effect of a behavioral school program on objective activity levels, while the program was judged as successful by teacher ratings [25]. A meta-analysis on the effect of methylphenidate on activity levels in children on the other hand found a significant reduction in objective activity levels after taking medication [26].

Differences in physical activity between children with and without ADHD have only been assessed in relatively small, clinical samples, not community samples. The aim of this study was to assess whether objectively measured physical activity in childhood (at age 7) over 7 days can predict ADHD symptoms (continuous) and ADHD diagnosis in adolescence (at age 14) in a large, representative, longitudinal UK population-based cohort study [27, 28].

## Methods

### Participants

Participants are from the Millennium Cohort Study (MCS), [29] a UK birth cohort study of 19,517 individuals born at the start of the millennium (Sept. 2000–Jan. 2002) who have been assessed at six sweeps to date: 9 months, 3, 5, 7, 11 and 14 years. Greater details of the study design, variables and attrition can be found at: https://cls.ucl.ac.uk/cls-studies/millennium-cohort-study/. Families were recruited via random sampling using all listed parents on the UK Child Benefit registers. [30] The sample is geographically clustered and is disproportionally stratified to allow areas of minority (e.g., ethnic minorities and disadvantaged areas) to be sufficiently represented. The MCS collects data on physical, socio-emotional, cognitive and behavioral development as the participants progress through childhood. Parents gave written informed consent for the participation in the study at each sweep. Cohort members gave verbal assent for wearing the accelerometers to collect the objective physical activity data.

Activity data were available from *N* = 6675 children, aged 7. Data for all four activity indices and SDQ ADHD data at ages 7 and 14 was available for *N* = 5251 children (descriptive data in Table 1. Parents reported that *N* = 74 children had been diagnosed with ADHD by the age of 14. Participants who had complete datasets had lower SDQ hyperactivity/inattention scores at age 7 and 14, lower moderate activity levels, and lower income (Table 2). It is therefore possible that our analyses underestimate the association between hyperactivity/inattention and activity level.

**Table 1:** Descriptive SDQ hyperactivity/inattention subscale and activity data

	*N*	SDQ hyp/inattention		Activity level in mins – Mean (SD)			
		Min–Max	Mean (SD)	Sedentary	Light	Moderate	Vigorous
Age 7	5251	0–10	3.22 (2.42)	391 (66)	281 (41)	43 (13)	20 (11)
Age 14	5251	0–10	2.93 (2.42)				

The table shows minimum (Min) and maximum (Max) values reached for the SDQ hyperactivity/inattention subscale at ages 7 and 14, and means and standard deviations (SD) of the SDQ hyperactivity/inattention scale at ages 7 and 14 and all activity levels at age 7

**Table 2:** Comparison between participants with complete and incomplete datasets

	Completeness	*N*	Mean	*t*	*p*
Age 7 SDQ	1.00	5251	3.22	− 5.69	< 0.001
	0.00	1120	3.67		
Age 14 SDQ	1.00	5251	2.93	− 3.90	< 0.001
	0.00	116	3.81		
Sedentary behavior	1.00	5251	390.61	− 0.75	0.455
	0.00	1257	392.21		
Light activity	1.00	5251	281.06	− 0.57	0.566
	0.00	1257	281.81		
Moderate activity	1.00	5251	42.83	− 3.83	<0.001
	0.00	1257	44.47		
Vigorous activity	1.00	5251	20.01	− 1.16	0.247
	0.00	1257	20.41		
Month of birth	1.00	5251	6.43	− 0.76	0.448
	0.00	1257	6.51		
Sex	1.00	5251	1.50	0.69	0.490
	0.00	242	1.48		
NVQ highest academic level (all sweeps)	1.00	4725	3.21	1.47	0.14
	0.00	142	3.07		
OECD equalized income	1.00	5190	400.09	9.29	< 0.001
	0.00	1242	332.91		

Independent *t *tests between participants with complete and incomplete datasets; 1 = complete dataset, 0 = incomplete dataset

### Measures

#### ADHD symptoms and diagnosis

The parent-rated subscale hyperactivity/inattention of the Strengths and Difficulties Questionnaire (SDQ; [31], an internationally used and validated screening questionnaire to assess mental and behavioral strengths and difficulties in 3–16-year-olds, was used at all measurement occasions to assess ADHD symptoms. The SDQ is widely used for measuring ADHD symptoms [32] and shows high correlations with other scales assessing ADHD symptoms as for instance the Conners Scale [33] or the Child Behaviour Checklist (CBCL; [34, 35]. The SDQ is better able to distinguish between children with and without ADHD than the CBCL with 118 items (11 for attention problems) [36]. The five-item inattention/hyperactivity subscale sums up the ratings of ADHD-related behavior and has good internal consistency (average Cronbach’s *α* = 0.87, maximum = 10 points). The items for the subscale are ‘restless, overactive, cannot stay still for long’, ‘constantly fidgeting or squirming’, ‘easily distracted, concentration wanders’, ‘thinks things out before acting’, ‘sees tasks through to the end, good attention span’. Every item is rated on a three point Likert scale: ‘not true’ (0), ‘somewhat true’ (1) and ‘certainly true’ (2). Positively worded items are reverse-scored. The possible range is 0–10 (*M* = 3.2 in the norm sample; [37]. Parents carried out the assessment. ADHD diagnosis was also reported by the parents using the following item: ‘Has a doctor or health professional ever told you that (sample child) had attention deficit hyperactivity disorder (ADHD)?’. *N* = 119 parents in the final sample gave information about ADHD medication at age 14. *N* = 42 children were taking ADHD medication at age 14, but 77 were not.

#### Actigraphy data

Activity was recorded using the Actigraph GT1M uni-axial-accelerometer (Actigraph, Pensacola, Florida) and the ActiLife Lifestyle Monitoring System software version 3.2.11 (Actigraph, Pensacola, Florida). Children were instructed to wear the accelerometer around the hip for seven consecutive days during waking hours. Sampling epochs were every 15 s; activity data and step count were recorded [38]. Four variables that were derived from the accelerometer data at age 7 included in the MCS dataset were used to predict ADHD symptoms at age 14 in this study: mean sedentary, light, moderate and vigorous activity. The variables reflect the mean time spent engaging in sedentary, light, moderate and vigorous activity across all valid days, as measured by acceleration (https://doc.ukdataservice.ac.uk/doc/8156/mrdoc/pdf/mcs6_2018_accelerometer.pdf).

### Statistical analysis

Several linear regression models were conducted in SPSS and compared, using *R*^2^ change. SDQ hyperactivity/inattention values at age 14 were predicted using linear regression with the four activity indicators as independent variables in model 1. Control predictors were entered gradually in consecutive models. In model 2, SDQ hyperactivity/inattention values at age 7 were entered as a control variable. Model 3 included sex, BMI at age 7 and 14, and month of birth (August = 12, September = 1), parental education across sweeps (parent education, excluding overseas education and “other” because it was unclear at what level they should be coded), parental income (OECD equivalised income) as an indicator of SES and ethnicity in addition. Due to the small number of parents giving information on medication intake for ADHD, we ran a separate, smaller model, predicting SDQ hyperactivity/inattention values at age 14 from the four movement parameters and hyperactivity/inattention values at age 7 (supplementary Table 1). Weights generated to account for both the sampling design of the Millennium Cohort Study and the smaller sample with accelerometry data were used to weight all analysis to obtain nationally representative estimates in all analysis.

Because of the low base rate of ADHD diagnosis in the sample (1.6%), a rare events logistic regression was conducted in R with ADHD diagnosis at age 14 as a dependent variable and the 4 activity indicators and sex as the independent variables.

## Results

The mean rating for SDQ hyperactivity/inattention declined from age 7–14 (see Table 1). The correlation between SDQ hyperactivity/inattention scales at age 7 and 14 was *R*(5251) = 0.57, *p* < 0.001. Correlations between SDQ hyperactivity/inattention at age 7 and the four activity indicators revealed a significant negative association between hyperactivity and sedentary activity *R*(6371) =   − 0.12, *p* < 0.001, and positive associations between hyperactivity and light *R*(6371) = 0.12, *p* < 0.001, moderate *R*(6371) = 0.15, *p* < 0.001 and vigorous *R*(6371) = 0.10, *p* < 0.001.

An ADHD diagnosis at age 7 (*N* = 68) was associated with significantly less sedentary behavior *t*(6476) =  − 2.55, *p* = 0.011, *d* =  − 0.33; more light *t*(6476) 2.18, *p* = 0.029, *d* = 0.28, moderate *t*(6476) = 2.19, *p* = 0.029, *d* = 0.27 and vigorous activity *t*(6476) = 3.25, *p* = 0.001, *d* = 0.35.

Regressions showed that sedentary behavior at age 7 negatively predicted hyperactivity/inattention at age 14 and that moderate activity at age 7 positively predicted hyperactivity/inattention at age 14 (Table 3). Sedentary behavior remained a significant predictor throughout all models. Additional significant predictors for hyperactivity/inattention at age 14 were hyperactivity/inattention at age 7, male sex, BMI at age 7 and 14, lower parental academic level, and lower SES. Having mixed or Asian ethnicity was a negative predictors for hyperactivity/inattention at age 14.

**Table 3:** Regression models predicting hyperactivity/inattention at age 14

	*R*^2^	*R*^2^ change	*F*	B	SE	*ß*	*t*	*p*	CI low	CI upper
Model 1	0.04		46.94***							
(constant)				2.09	0.43		4.90	** < 0.001**	1.25	2.92
Activity										
Sedentary				− 0.002	0.001	− 0.07	− 4.00	** < 0.001**	− 0.004	− 0.001
Light				0.003	0.001	0.05	2.64	**0.008**	0.001	0.005
Moderate				0.02	0.004	0.12	5.04	** < .001**	0.01	0.03
Vigorous				0.004	0.005	0.02	0.87	0.39	− 0.005	0.13
Model 2	0.34	.01***	477.07***							
(constant)				1.15	.36		3.19	**0.001**	0.44	1.85
SDQ Age 7				0.55	0.01	0.55	45.94	** < 0.001**	0.53	0.57
Activity										
Sedentary				− 0.002	0.001	− 0.04	− 3.12	**0.002**	− 0.003	− 0.001
Light				< 0.001	0.001	0.003	0.22	0.828	− 0.001	0.002
Moderate				0.01	0.004	0.07	3.49	** < 0.001**	0.005	0.019
Vigorous				0.001	0.004	0.006	0.37	0.72	− 0.006	0.009
Model 3	0.35	.004***	159.06***							
(constant)				3.35	.48		7.00	** < 0.001**	2.41	4.29
Age 7 SDQ				0.52	0.01	0.52	40.91	** < 0.001**	0.49	0.54
Sex				− 0.42	0.07	− 0.09	− 6.56	** < 0.001**	− 0.55	− 30
Age 7 BMI				− 0.08	0.02	− 0.08	− 4.20	** < 0.001**	− 0.12	− 0.05
Age 14 BMI				0.03	0.01	0.05	2.66	**0.008**	0.008	0.05
Month of birth				0.01	0.01	0.01	0.87	0.382	− 0.009	0.02
Highest academic level				− 0.12	0.03	− 0.06	− 4.45	** < 0.001**	− 0.17	− 0.07
Income				− 0.001	< 0.001	− 0.06	4.39	** < 0.001**	− 0.001	< 0.001
Ethnicity										
Mixed				− 1.15	0.35	− 0.04	− 3.33	**0.001**	− 1.83	− 0.47
Asian				− 56	0.14	0.05	− 4.02	** < 0.001**	− 0.83	− 0.28
Black				0.04	0.18	0.003	0.22	0.823	− 0.31	0.39
Other				0.18	0.28	0.008	0.63	0.531	− 0.37	0.73
Activity										
Sedentary				− 0.001	0.001	− 0.04	− 2.62	**0.009**	− 0.003	− 0.0003
Light				− 0.001	0.001	0.01	0.68	0.496	− 0.001	0.002
Moderate				0.01	0.004	0.03	1.36	0.173	− 0.002	0.01
Vigorous				0.003	0.004	0.01	0.71	0.476	− 0.005	0.1

Linear regression models show that all activity levels at age 7, apart from vigorous behavior, significantly predict ADHD symptoms at age 14. When controlled for ADHD symptoms at age 7, sex, BMI, SES, month of birth and ethnicity, less sedentary behavior remains as a significant predictor of ADHD symptoms at age 14. Significant *p* values are in bold

### Predicting ADHD diagnosis from accelerometer data

A rare events logistic binary regression showed that ADHD diagnosis at age 14 was significantly predicted by less sedentary behavior at age 7 and male sex (Table 4).

**Table 4:** Regression predicting ADHD diagnosis at age 14

	B	SE	*z*	*P*
Sedentary activity	− 0.008	0.003	− 2.13	**0.033**
Light activity	− 0.001	0.01	− 0.13	0.901
Moderate activity	− 0.01	0.02	− 0.34	0.736
Vigorous activity	0.04	0.02	1.67	0.094
Sex	− 1.41	0.39	− 3.63	**0.001**
Intercept	0.03	2.58	0.01	0.99

A rare events logistic regression shows that less sedentary behavior and sex are significant predictors of an ADHD diagnosis at age 14. Significant *p* values are in bold

## Discussion

The results show that lower levels of sedentary activity during the day at age 7 can significantly predict symptoms of ADHD at age 14 over and above parent report. Furthermore, lower levels of sedentary behavior at age 7 can significantly predict an ADHD diagnosis at age 14. Approximately, 15–70% of children with ADHD continue to display ADHD symptoms in adolescence [39, 40]. It was previously shown in 116 patients with an ADHD diagnosis that ADHD symptoms in adolescence can be predicted by parent-rated hyperactivity/impulsivity and inattention in childhood, comorbidities, SES, and mean activity level [41]. Our results confirm these findings in 5251 children from a large prospective national cohort. In contrast, studies predicting ADHD in childhood from objective movement parameters in infancy did not find this effect [20, 23]. It is possible that these studies did not find this effect because they did not tap into movements long enough to measure reliably, or because they focused on activity, rather than sedentary behavior. Interestingly, our results reveal that lower levels of sedentary activity at age 7 were the main predictor for ADHD at age 14, rather than higher levels of vigorous activity. This effect became larger when controlling for intake of ADHD medication in a subsample. Taken together, objective movement parameters can explain approximately 4% of parent-rated ADHD symptoms 5 years later. The effect size appears rather small when compared to the 30% of explained variance in parent-rated ADHD symptoms at age 14 by parent-rated ADHD symptoms at age 7. However, activity measures are a very different type of assessment from parent reports and explain independent variance over and above parent report. It may be possible to use objective activity data, particularly regarding sedentary activity, to help inform ADHD diagnoses.

A test has already been developed, the QbTest, that can augment clinical diagnosis by assessing performance on a CPT and combining that with activity levels assessed via infrared camera. The test has been shown to differentiate between ADHD and autism spectrum disorder [8] and can help with diagnostic decision making [9]. Compared to the QbTest, activity data were collected in a natural setting in this cohort, i.e., at home, on seven consecutive days. It would be useful to directly compare short activity measures, such as the QbTest, and activity data collected over longer time periods. One important criterion for an ADHD diagnosis is that symptoms have to occur trans-situationally. Activity data collected over a week may give a more accurate picture regarding activity level and may increase sensitivity and specificity if used for diagnosis. However, activity levels over a long period of time and a short interval in a clinical setting should be compared to see if long measurements are significantly different. The finding that sedentary behavior is more important than moderate or high activity to predict symptoms could also be used to enhance the diagnostic value of activity measures. A limiting factor is that actigraphy specifically targets hyperactivity/impulsivity. Inattentive types might not be identified using this method and predictions of our models may have been enhanced if only items assessing hyperactivity were used. This may also explain why sex was a significant predictor, as females present with the inattentive type more often than males and males meet criteria for the combined type more often than females and tend to display more externalizing symptoms [42].

Other significant predictors of hyperactivity/inattention in adolescents were higher parent-reported levels of hyperactivity/inattention at age 7, being male, SES (parent income and academic level), BMI and ethnicity. While childhood ADHD symptom severity as a relevant predictor for adulthood ADHD symptoms has been well established [41, 43], evidence on the relationship between symptoms of ADHD and SES is somewhat mixed. Some evidence suggests that a lower SES is associated with more symptoms of ADHD [44] persistence of ADHD [41, 45, 46] and better treatment response [47], while other evidence indicated that there was no simple predictive relationship between the two [48–50]. Our result showed that both lower parent income and lower academic level significantly predicted higher symptoms of ADHD in adolescence. However, the effects were small, particularly for parent income.

Children of mixed and Asian ethnicity had lower parent-reported hyperactivity levels than white children. Several studies haves shown that nonwhite children were less likely to be diagnosed with ADHD than white children [51–54], whereas other studies have shown no differences between black and white children [55] or higher rates in black children [56] but lower rates in Hispanic children in the USA [57]. However, all the above studies were conducted in the USA and a recent study indicated that the differences between white and black children are likely due to an underidentification of ADHD in black children rather than lower prevalence rates, but that Hispanic children were indeed less likely to meet ADHD diagnostic criteria [54]. One possibility for differences in parent-rated symptoms is that perception of hyperactivity by the parents is culturally influenced [58, 59].

Interestingly, a lower BMI at age 7 predicted hyperactivity/inattention at age 14, but a higher BMI at age 14 was associated with higher hyperactivity/inattention levels at age 14. ADHD has been associated with disordered eating (problematic eating behavior that does not meet diagnostic criteria) and eating disorders [60, 61]. Disinhibited eating behaviors may be related to inattention and deficits in awareness of internal hunger signals [62]. A review concluded that children with ADHD were at increased risk for disordered eating, while adolescents were at increased risk for having and eating disorder or disordered eating [61]. ADHD has been linked to a higher BMI in adults [63] and children from age 9 [64], but the trajectory of the relationship between BMI and hyperactivity from early childhood to adulthood is not well researched.

Regarding different activity levels at age 7, the data showed that children with an ADHD diagnosis were more active than children without an ADHD diagnosis. This is in line with previous studies [11–13, 16, 18, 65–67]. Differences between predominantly inattentive and combined ADHD subtypes were not found [16]. Again, our results show that the main difference lies in sedentary behavior. Children with an ADHD diagnosis spent less time in sedentary behavior than the control group. Moreover, children with an ADHD diagnosis spent more time with moderate activity levels than children without a diagnosis. This finding was further corroborated by smaller, but significant associations between sedentary and moderate behavior with parent-rated hyperactivity at age 7. Even though higher levels of moderate activity are also related to current and future symptoms of ADHD, it is interesting that the main difference between hyperactive children and children who are not hyperactive, or less so, appears to lie in lower levels of sedentary activity.

A limitation of the current study is that common comorbidities that can affect activity levels, such as tics, could not be taken into account. Moreover, participants who had complete datasets had lower SDQ hyperactivity/inattention scores at age 7 and 14, lower moderate activity levels, and lower income. It is therefore possible that our analyses underestimate the association between hyperactivity/inattention and activity level.

In conclusion, hyperactivity and ADHD diagnosis at age 14 can be significantly predicted by objective activity levels at age 7, particularly by lower sedentary activity levels. Measuring sedentary activity levels during waking hours could be further developed to aid in the early identification and diagnosing of ADHD.

## Electronic supplementary material

Below is the link to the electronic supplementary material.

Supplementary file1 (DOCX 15 kb)
