# Supplementary material for: Predicting ADHD symptoms and diagnosis at age 14 from objective activity levels at age 7 in a large UK cohort
Source: Eur Child Adolesc Psychiatry. 2020 Jun 6;30(6):877–84. doi: 10.1007/s00787-020-01566-9 (PMC8140967; doi:10.1007/s00787-020-01566-9)
Supplement: Supplementary file 1 — Supplementary file1 (DOCX 15 kb) [file 787_2020_1566_MOESM1_ESM.docx]

| *Supplementary Table 1.* Regression predicting hyperactivity, controlled for medication | | | | | | | | |  |  |
| --- | --- | --- | --- | --- | --- | --- | --- | --- | --- | --- |
|  | **R^2^** |  | ***F*** | **B** | **SE** | **ß** | ***t*** | ***p*** | ***CI low*** | ***CI***  ***upper*** |
| **Model 1** | .18 |  | 3.933*** |  |  |  |  |  |  |  |
| (Constant) |  |  |  | 11.57 | 2.48 |  | 4.68 | **<.001** | 6.67 | 16.47 |
| ADHD medication |  |  |  | -1.03 | .44 | -.22 | -2.34 | **.021** | -1.90 | -.16 |
| Age 7 SDQ |  |  |  | .21 | .08 | .24 | 2.61 | **.01** | .05 | .36 |
| Activity:  Sedentary |  |  |  | -0.008 | 0.003 | -0.26 | -2.42 | **.017** | -.015 | -.001 |
| Light |  |  |  | < 0.001 | 0.05 | <0.001 | -.001 | .999 | -.01 | .02 |
| Moderate |  |  |  | -0.02 | 0.03 | -0.11 | -.702 | .484 | -.07 | .04 |
| Vigorous |  |  |  | 0.009 | 0.03 | 0.05 | 0.34 | .734 | -.04 | .06 |
|  |  |  |  |  |  |  |  |  |  |  |
